# Comparison of Retinal Nerve Fiber Layer Thickness *In Vivo* and Axonal Transport after Chronic Intraocular Pressure Elevation in Young versus Older Rats

**DOI:** 10.1371/journal.pone.0114546

**Published:** 2014-12-11

**Authors:** Carla J. Abbott, Tiffany E. Choe, Claude F. Burgoyne, Grant Cull, Lin Wang, Brad Fortune

**Affiliations:** Discoveries in Sight Research Laboratories, Devers Eye Institute and Legacy Research Institute, Legacy Health, Portland, Oregon, United States of America; Hanson Institute, Australia

## Abstract

**Purpose:**

To compare in young and old rats longitudinal measurements of retinal nerve fiber layer thickness (RNFLT) and axonal transport 3-weeks after chronic IOP elevation.

**Method:**

IOP was elevated unilaterally in 2- and 9.5-month-old Brown-Norway rats by intracameral injections of magnetic microbeads. RNFLT was measured by spectral domain optical coherence tomography. Anterograde axonal transport was assessed from confocal scanning laser ophthalmolscopy of superior colliculi (SC) after bilateral intravitreal injections of cholera toxin-B-488. Optic nerve sections were graded for damage.

**Results:**

Mean IOP was elevated in both groups (young 37, old 38 mmHg, p = 0.95). RNFL in young rats exhibited 10% thickening at 1-week (50.9±8.1 µm, p<0.05) *vs.* baseline (46.4±2.4 µm), then 7% thinning at 2-weeks (43.0±7.2 µm, p>0.05) and 3-weeks (43.5±4.4 µm, p>0.05), representing 20% loss of dynamic range. RNFLT in old rats showed no significant change at 1-week (44.9±4.1 µm) *vs.* baseline (49.2±5.3 µm), but progression to 22% thinning at 2-weeks (38.0±3.7 µm, p<0.01) and 3-weeks (40.0±6.6 µm, p<0.05), representing 59% loss of dynamic range. Relative SC fluorescence intensity was reduced in both groups (p<0.001), representing 77–80% loss of dynamic range and a severe transport deficit. Optic nerves showed 75–95% damage (p<0.001). There was greater RNFL thinning in old rats (p<0.05), despite equivalent IOP insult, transport deficit and nerve damage between age groups (all p>0.05).

**Conclusion:**

Chronic IOP elevation resulted in severely disrupted axonal transport and optic nerve axon damage in all rats, associated with mild RNFL loss in young rats but a moderate RNFL loss in old rats despite the similar IOP insult. Hence, the glaucomatous injury response within the RNFL depends on age.

## Introduction

Glaucoma is a chronic, progressive optic neuropathy and a leading cause of blindness [1], however much is still unknown regarding its pathophysiology. The degeneration and drop-out of retinal ganglion cell (RGC) axons and somata that leads to permanent vision loss is usually attributed to an initial injury to axons within the optic nerve head (ONH) [2], [3]. Axonal transport blockade in RGC axons within the ONH is thought to be an early and critical pathological event in this sequence of glaucomatous damage [3]–[21]. Reversal of axonal transport blockade in acute intraocular pressure (IOP) elevation models (generally <8 hours) by IOP normalization allows axon recovery and prevents RGC death [5], [22], [23]. However, the process by which an acute but reversible axonal transport deficit becomes a pathological contributor to axonal injury remains unclear. Repeated, intermittent IOP insults [24]–[28] or longer-duration chronic IOP elevation where the IOP–time integral reaches above a certain threshold [29] may both play a role in triggering mechanisms leading to chronic changes and RGC death. Older age is also known to be a critical risk factor for glaucoma [30], with glaucoma prevalence increasing sharply with age [1], [31] and most longitudinal studies finding that age is an important risk factor for glaucoma progression [32]–[35]. A previous study has also found that axonal transport deficits are worse in older rats with experimental glaucoma as compared with younger rats exposed to a similar level of IOP elevation [17]. It seems likely there is an interaction between axonal transport, RGC injury and age, such that axonal transport blockade may occur more quickly and severely for a given IOP insult in older as compared with younger animals. With recent advances in technical capabilities for *in vivo* imaging, it is also possible to compare the timing of deficits in axonal transport with the structural integrity of the retinal nerve fiber layer, such as the thickness of this tissue layer, a parameter used commonly in the clinical management of glaucoma.

In this study, we investigated whether chronic experimental elevation of IOP for 3-weeks induced by microbead injection would produce a different response between young and old rats in terms of the severity of axonal transport blockade and the amount of permanent RGC injury, as characterized by *in vivo* retinal nerve fiber layer thickness (RNFLT) changes and optic nerve axon damage. The approach of monitoring longitudinal *in vivo* changes in RNFLT also enabled evaluation of the sequence of RNFLT changes due to chronic IOP elevation and comparison to the course of axonal transport and optic nerve axon injury at the 3-week endpoint. To our knowledge, this is the first comparison of *in vivo* RNFLT with axonal transport blockade and optic nerve axon damage.

## Methods

### Subjects

The subjects of this study were 28 adult male Brown-Norway rats (*Rattus norvegicus*; Charles River Laboratories Inc. Wilmington, MA); N = 12 young rats with unilateral IOP elevation, N = 12 old rats with unilateral IOP elevation and N = 4 young naïve rats. Rats in the young age-group were aged 2 to 3 months and weighed 203 to 226 g at baseline. Rats in the older age-group were retired breeders aged 9.5 to 10.5 months and weighed 330 to 397 g at baseline. Rats were maintained under a 12-hour light/12-hour dark cycle with normal rat chow and water available *ad libitum*. All experimental methods and animal care procedures adhered to the Association for Research in Vision and Ophthalmology's Statement for the Use of Animals in Ophthalmic and Vision Research, were carried out in strict accordance with the recommendations in the Guide for the Care and Use of laboratory Animals of the National Institutes of Health and were approved and monitored by the Institutional Animal Care and Use Committee (IACUC) at Legacy Health (USDA license 92-R-0002 and OLAW assurance A3234-01). All efforts were made to minimize suffering.

### Anesthesia

For all injection and imaging procedures, animals were anesthetized with an intramuscular injection of a rodent cocktail containing ketamine (55 mg/kg, Ketaset; Fort Dodge Animal Health, Fort Dodge, IA), xylazine (5 mg/kg, AnaSed; Lloyd, Inc., Shenandoah, IA), and acepromazine maleate (1 mg/kg; Vedco, Inc., St. Joseph, MO). This dosage provided anesthesia for 45–90 minutes. Half of the original dose was used for repeated injections as necessary. Body temperature was maintained with a heat mat and lactated Ringers solution (Baxter Healthcare Corporation, Toronto, Canada) was injected subcutaneously to maintain hydration.

### Chronic IOP Elevation Protocol

Injection of microbeads and viscoelastic both separately and in combination into the anterior chamber have been well-established in the literature as techniques to experimentally increase IOP for extended (chronic) periods of days to weeks, although multiple injections are often required especially for periods greater than 2-weeks [17], [36]–[46]. The location of microbeads within the anterior chamber can be difficult to control with latex microbeads, which tend to coalesce and pool into the inferior chamber angle and adhere to the lens or posterior cornea in the pupillary zone, thus compromising optical media for retinal imaging. Hence, for this study we chose to use ferro-magnetic microbeads in a manner similar to that described in a previous study by Samsel et al [46]. One advantage of this approach is that a magnet can be used to direct the paramagnetic microbeads away from the optical axis and to spread their distribution into all aspects of the anterior chamber angle.

Unilateral intracameral injections of 10 µm diameter ferro-magnetic microbeads (#109319-10; Corpuscular Inc., NY), suspended 30 mg/ml in a sterile balanced salt solution (BSS; Alcon, TX), were performed to induce chronic IOP elevation, as used by Samsel et al [46]. Prior to injection, the microbeads were progressively washed in 10xTris buffer, distilled water and sterile BSS for 48 hours under a laminar flow hood. Topical anesthetic (0.5% proparacaine hydrochloride, Alcon Laboratories Inc.) and topical povidone-iodine (10%, Walgreens, IL; diluted to 5% with BSS) were instilled on the experimental eye immediately prior to injection. The microbeads were injected through peripheral cornea via a 30.5 G needle (Becton Dickinson, NJ) and Hamilton syringe using a tunneling technique to promote self-sealing. The beads were manipulated into the anterior chamber angle with a handheld magnet (0.5 to 0.6 Tesla at surface) to reduce the outflow of aqueous humor through the trabecular meshwork and Schlemm's canal. The injection hole was plugged with viscoelastic (Healon-5, Abbott Medical Optics, IL) to minimize leakage of aqueous, microbeads and vehicle out of the anterior chamber and to minimize post-injection hypotony. Topical antibiotic ointment (neomycin, polymyxin B sulfates and dexamethasone, Falcon Pharmaceuticals Ltd, Fort Worth, Texas) and ocular lubricant (Celluvisc, Allergan, CA) were applied to both eyes after the injection. The fellow eye of each animal served as a non-injected control.

One to three injections of microbeads were required in order to maintain elevated IOP over the 3-week follow-up period. The number of injections required to maintain IOP elevation was not significantly different between age groups (young 2.42±0.51, old 1.92±0.79, p = 0.08 unpaired t-test) The quantity of microbeads injected was based on the Samsel et al. methods [46] and refined with pilot studies. For the first injection, 10 µl of the bead solution (∼5.4×10^5^ beads) was injected and spread into 80% of the anterior chamber angle. If IOP in conscious rats at follow-up did not achieve or was not maintained ≥30 mmHg, then a second or third injection of 5 µl bead solution (∼2.7×10^5^ beads) was performed, covering nearly 100% of the anterior chamber angle. Microbeads were re-suspended in solution with a vortex (Thermolyne, Barnstead International, IA) or ultrasonic stirrer (1210 Bransonic, Branson Ultrasonics, CT) immediately prior to injection.

### Intraocular Pressure (IOP) Measurements

IOP measurements were taken using a rodent tonometer (Tonolab). Rats were trained in having awake IOP measurements performed for four consecutive days prior to beginning baseline measures since behavioral training has been shown to reduce IOP over the first 4–6 days [47]. All IOP recordings were measured in conscious rats under topical anesthesia as the average of five consecutive measurements. Baseline and follow-up IOP measures were taken within a 4-hour time window so as to be minimally affected by diurnal variation.

### In Vivo SD-OCT Measurements

*In vivo* peripapillary RNFLT and total RT were measured longitudinally using spectral domain OCT (SD-OCT, Spectralis, Heidelberg Engineering GmbH, Germany) as previously described [23], [48]. Topical anesthesia and mydriasis (tropicamide 0.5%, Alcon Laboratories Inc.; phenylephrine 2.5%, Bausch and Lomb Pharmaceuticals Inc., Tampa, FL) were instilled, custom rigid gas-permeable contact lenses inserted, and rats placed on a custom-built imaging stage. Average and sectoral (superior, inferior, nasal, temporal) peripapillary RNFLT and RT values were determined from circular B-scans (12° diameter) centered on the optic disc. The digital axial resolution was 3.9 µm. Each B-scan was comprised of 1536 A-scans and an average of 100 sweeps using automatic real-time eye-tracking software to reduce speckle noise. Follow-up scans utilized the eye-tracking software and were identical to the scan position at baseline. In some cases, changes in optical quality prevented the eye-tracking software from engaging for follow-up scan mode. In such cases, a manual alignment procedure was used to align with the position at baseline as best as possible. Manual retinal layer segmentations were performed with custom software to edit the instrument's native segmentations as previously described [48]. The editing was performed by a single trained observer and was required when the native segmentation identified the wrong features in the scan (particularly for the RPE/BM complex, which is common for scans of rat eyes) [48]. In this study, a single baseline measurement of RNFLT was obtained as the repeatability of this method, including scan acquisition and image segmentation, has been evaluated previously in three separate, published cohorts [23], [48], [49].

### Anterograde Axonal Transport Assay

Anterograde axonal transport within RGCs was assessed with the tracer cholera toxin b-subunit conjugated to AlexaFluor488 (CTB; dissolved in sterile PBS) as previously described [23], [50]. Briefly, the ocular fundi were imaged *in vivo* by confocal scanning laser ophthalmoscopy in fluorescence mode (CSLO-FL; Spectralis HRA, Heidelberg Engineering GmbH, Germany) 24 hours after bilateral intravitreal injection of 2 µl 1% CTB, to confirm successful injections. Rats were then overdosed with an intraperitoneal injection of pentobarbital sodium and phenytoin sodium (0.7–1.4 ml/kg; Euthasol Solution, Virbac Animal Health Inc., Fort Worth, Texas). The eyes were enucleated, and the rats transcardially perfused with 0.1 ml heparin sodium (10,000 USP Units/ml, APP Pharmaceuticals) followed by 125 ml of cold 4% paraformaldehyde in 0.5 M phosphate buffer (PB, pH 7.35). The retinas and brain were dissected and immersion fixed in 4% paraformaldehyde in 0.5 M PB for 30 minutes. Whole-mounted retinas were imaged with fluorescence microscopy (x5 air objective; Leica DMRXE, Germany) with filter set #513808 (FITC; 450–490 nm excitation, 515 nm long pass emission; Chroma) to additionally confirm successful injections.

Axonal transport was assessed from post-mortem CSLO-FL (Spectralis HRA) of the superior colliculi with an additional +25 D lens mounted to the camera objective [50]. The BluePeak blue laser (488 nm) autofluorescence imaging mode with standard contrast (not normalized) and a fixed camera sensitivity (set to 90) was used with 100 frames averaged. The relative fluorescence intensity was compared between experimental and fellow control superior colliculi in ImageJ software (NIH) with a colliculus-shaped polygon. Approximately 95% of axons cross at the chiasm in pigmented rats [51], [52], so the experimental superior colliculus is of opposite laterality to the experimental eye.

### Optic Nerve Grading Analysis

Optic nerve segments approximately 1 mm from the optic disc were dissected, immersed in 5% Sorensen's phosphate buffered gluteraldehyde for 90 minutes, post-fixed in 1% osmium tetroxide for 3 hours, dehydrated in an ethyl alcohol and acetone series, and embedded in epoxy resin (Poly/Bed 812, Polysciences Inc., PA) for sectioning. Cross-sections (1 µm thick) of optic nerve were cut with a diamond knife on an ultramicrotome (RMC Ultramicrotome MT6000; Boeckeler Instruments Inc., AZ) and stained with a myelin stain (2% p-phenylenediamine in 50% ethanol). Micrographs were acquired as previously described [53], using a digital camera (QICAM 12-bit, QImaging, Surrey, BC, Canada) attached to a Leica DM IRB inverted microscope with a 100x oil objective, imaging software (Qcapture, Qimaging) and a computer-controlled (X-Y-Z) stage (Applied Scientific Instrumentation Inc., Eugene OR). Micrographs were taken with a 15% overlap in x and y directions and montaged in custom software to display the entire nerve cross-section. Optic nerve montages were graded by a trained, masked observer and assigned a damage grade from 1 to 5 based on an established grading scheme [54], [55]. Grade 1 represents a normal optic nerve with only a few degenerating axons, grade 2 indicates focally degenerating axons and some axonal swellings, grade 3 shows numerous degenerating axons and axonal swellings starting to spread from a focal area, grade 4 represents numerous degenerating and swollen axons throughout the entire nerve and grade 5 is degenerating swollen axons across nearly the entire nerve and signs of extensive gliosis such as thickened septae, with few to no normal axons present (i.e. nearly 100% axon damage).

### Experimental Design

Rats were assigned by age to the 2-month old group (‘young’, n = 12) or the 9-month old group (‘old’, n = 12). Baseline IOP measurements in awake, trained rats were averaged over five consecutive days. Follow-up awake IOP data was collected every second day from day-1 after the initial injection of microbeads until day-21. SD-OCT measurements were taken at baseline and at 1-, 2-, and 3-weeks follow-up. Sometimes SD-OCT images were unable to be taken due to media opacity (usually corneal edema following microbead injection, but also due to microbeads stuck to the corneal endothelium or anterior lens capsule along the visual axis). Hence, the final numbers included in the SD-OCT analysis are: 2-month group n = 12 at baseline, n = 11 at week-1, n = 8 at week-2, n = 7 at week-3; 9-month group n = 9 at baseline (since 3 rats did not have SD-OCT data for any follow up time point), n = 6 at week-1, n = 8 at week-2, n = 7 at week-3. The anterograde axonal transport assay and animal euthanasia was performed at 3-weeks in ten 2-month-old and ten 9-month-old rats. One 2-month old rat underwent axonal transport assay and was sacrificed at 2-weeks due to neovascularization of the iris (large hyphema present) and one 9-month old rat underwent axonal transport assay and was sacrificed at 1-week due to severe corneal edema presumably from loss of endothelial cells from a poor intracameral injection. One rat in each group died on day-20 prior to performing the axonal transport assay. All 20 rats assayed at 3-weeks had successful intravitreal CTB injections (good uptake of CTB across central retina). Hence, n = 10 rats in each age group were included in the axonal transport datasets. For proximal optic nerve axon damage grading, both optic nerves in the 11 rats in each age group that were euthanized at 3-weeks were included in analysis (i.e. total 22 rats). A further naïve normal control group (seven nerves from four 2-month old rats) was used just for optic nerve axon damage grading and did not undergo IOP elevation.

### Statistics

Two-way ANOVAs with Bonferroni-corrected post hoc tests were used to analyze IOP, anterograde axonal transport, RNFLT, RT and dynamic range data. A one-way Kruskal-Wallis ANOVA with Dunn's post hoc tests was performed on optic nerve grading data. All statistical analysis was done using commercial software (Prism, Version 5, GraphPad Software, Inc., CA).

## Results

### IOP

**Figure 1:**
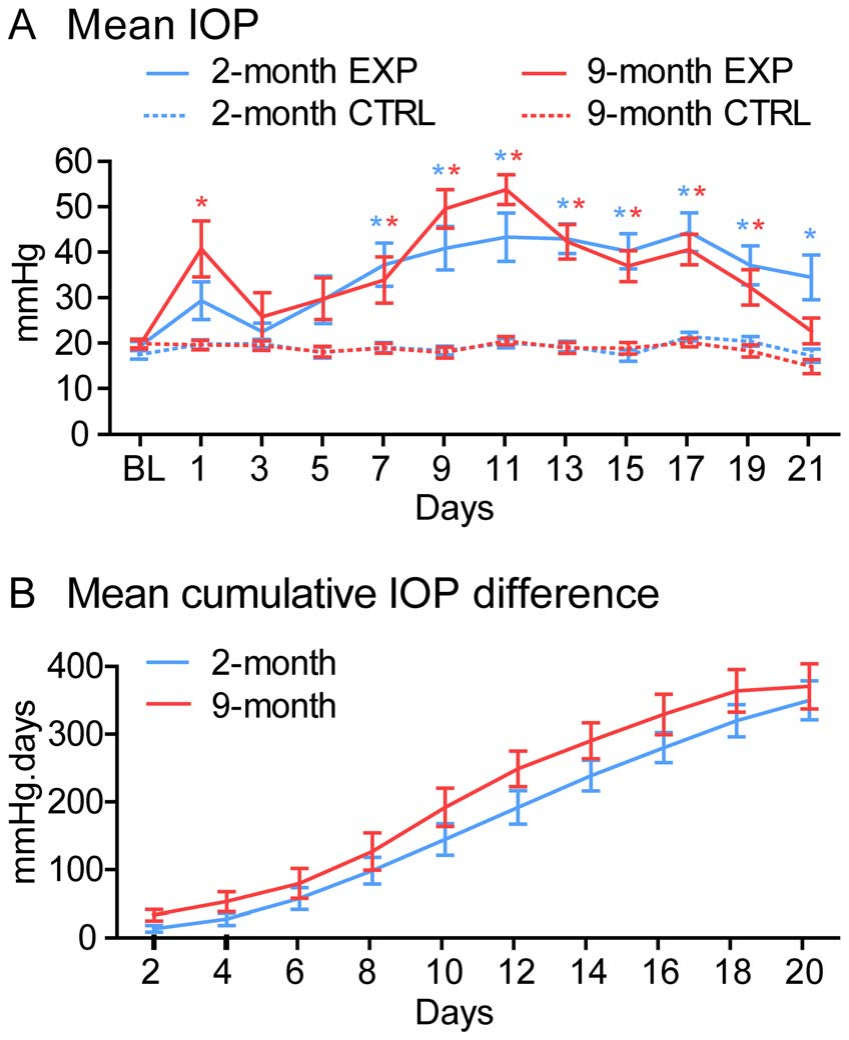
IOP elevation. (A) Mean IOP versus time. There was a significant difference between experimental and fellow control eyes within each age group, but not between age groups. (B) Mean cumulative inter-eye IOP difference versus time. Cumulative IOP difference increased as expected with time, but there was no difference between age groups. Error bars  =  SEM. * indicates p<0.05 when comparing experimental and fellow control eyes (Bonferroni). Abbreviations: EXP  =  experimental eyes, CTRL  =  fellow control eyes, BL  =  baseline.

Mean IOP in experimental eyes showed a small increase at day-1 follow-up, followed by recovery at day-3 before increasing again after further microbead injections and remaining elevated between day-7 and day-21 (Fig. 1A). There was no difference between age groups in experimental eye mean IOP over the 3-weeks (p = 0.68), although the 9-month group trended higher at 1-day and 10-days post injection. The experimental eye IOP averaged across all post-injection time points was 36.5±4.3 (SD) mmHg in 2-month old rats and 37.5±5.3 in 9-month old rats (p = 0.95, Mann-Whitney). Mean IOP in all fellow control eyes remained steady across the entire follow-up period. Peak IOP (the highest IOP observed across any time point for each rat) in experimental eyes was 61.2±6.2 mmHg in 2-month old rats and 61.9±12.1 in 9-month old rats (p = 0.34, Mann-Whitney). The mean cumulative inter-eye IOP difference increased for both age groups over time (p<0.001; Fig. 1B). There was no difference between age groups by post-hoc tests of each time point (p>0.05), although the 9-month group trended slightly higher.

### RNFL Thickness

**Figure 2:**
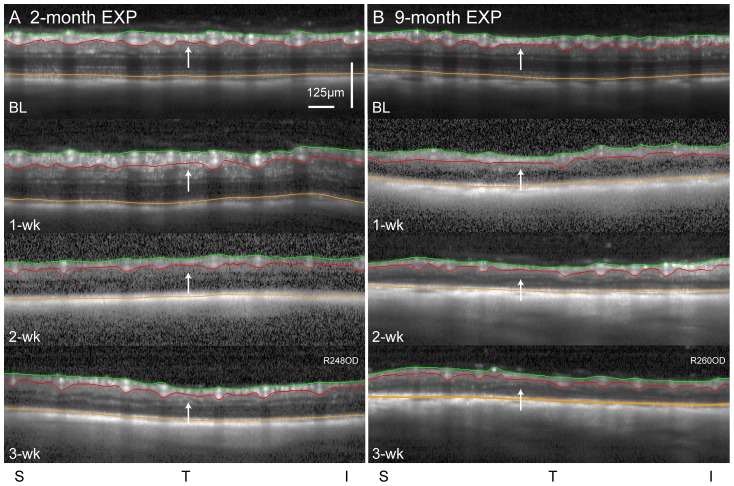
Peri-papillary SD-OCT B-scans. B-scans are shown for a single representative rat from the 2-month (A) and 9-month (B) age groups at baseline and at 1-, 2- and 3-weeks (wk) after chronic, unilateral IOP elevation. Segmentations of the inner limiting membrane (green), outer border of the nerve fiber layer (red) and Bruch's membrane/retinal pigment epithelium complex (orange) are marked, and were used to derive longitudinal measures of peripapillary RNFLT (green to red) and RT (green to orange). In the 2-month eye, arrows point to a region of the RNFL that shows initial thickening at 1-week, followed by thinning at 2- and 3-weeks. In the 9-month eye, arrows show a region of RNFL undergoing progressive thinning beginning at 1-week. Abbreviations: EXP  =  experimental eye, S  =  superior, T  =  temporal, I  =  inferior. Scale applies A-F.

**Figure 3:**
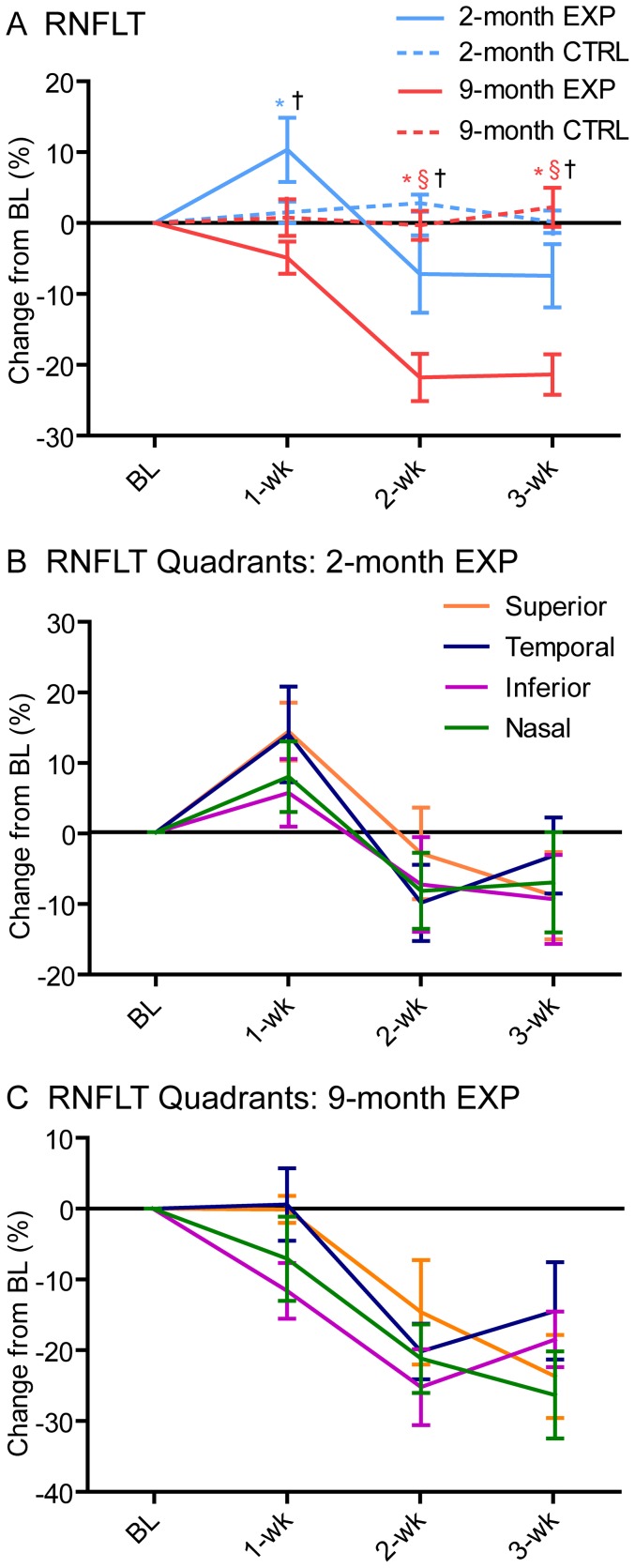
Percent change in RNFLT relative to baseline. (A) Change in total peripapillary RNFLT for experimental and fellow control eyes in both age groups. The 2-month group experimental eyes exhibit initial RNFL thickening, followed by thinning, while the 9-month group experimental eyes only exhibit thinning and do so to a greater extent than the younger eyes. (B) Change in RNFLT across quadrants for 2-month experimental eyes. (C) Change in RNFLT across quadrants for 9-month experimental eyes. There was no significant difference between quadrants in either age group although the superior and temporal quadrants trended thicker than inferior and nasal quadrants at earlier time points. Error bars  =  SEM. * indicates p<0.05 when compared to baseline, § indicates p<0.05 when compared to fellow control eyes, † indicates p<0.05 when comparing 2-month and 9-month experimental eyes (Bonferroni). Legend in B also applies to C. Abbreviations: EXP  =  experimental eyes, CTRL  =  fellow control eyes, BL  =  baseline.

Fig. 2 provides a representative example of longitudinal RNFLT measurements derived from peripapillary, circular SDOCT B-scans at baseline and at 1-, 2- and 3-weeks after chronic IOP elevation in the experimental eye of one individual 2-month and one individual 9-month animal. The aggregate results for longitudinal RNFLT changes are plotted for each study group in Fig. 3A as percent change from baseline. There is a difference in the course and ultimate extent of RNFL thinning seen between age groups. The 2-month experimental eyes undergo initial *thickening* at 1-week (50.9±8.1 µm, 10.3±15.0%, p<0.05) compared to baseline (46.4±2.4 µm), which is then followed by mild thinning at 2-weeks (43.0±7.2 µm, −7.2±15.4%, p>0.05) and 3-weeks (43.5±4.4 µm, −7.4±11.8%, p>0.05). In contrast, the 9-month experimental eyes show early signs of RNFL thinning from their baseline average of 49.2±5.3 µm by 1-week (44.9±4.1 µm, −4.9±5.6%, p>0.05), which progresses by 2-weeks (38.0±3.7 µm, −21.8±9.4%, p<0.01) and 3-weeks (40.0±6.6 µm, −21.4±7.6%, p<0.05). These changes in RNFLT are consistent with the amount of change expected to achieve significance for a sample of this size based on previous repeatability analysis of three separate cohorts of rats [23], [48], [49]. Furthermore, studies of clinical test-retest repeatability of RNFLT in humans converge on a value of about ±5 µm (±5%) [56]–[58]. Therefore, the changes found in the RNFLT parameter in the rat eye are likely to be meaningful since they are beyond 5%.

There was no significant change from baseline (2-month 46.5±2.3 µm; 9-month 48.9±5.8 µm) in RNFLT of the fellow control eyes in either age group at any of the follow-up time points (all p>0.05). RNFLT values in the fellow control eyes of both age groups remain nearly constant across the entire follow-up period. There were no differences between quadrants in the magnitude of RNFLT changes in either 2-month (Fig. 3B) or 9-month (Fig. 3C) experimental eyes (all p>0.05).

### Retinal Thickness

**Figure 4:**
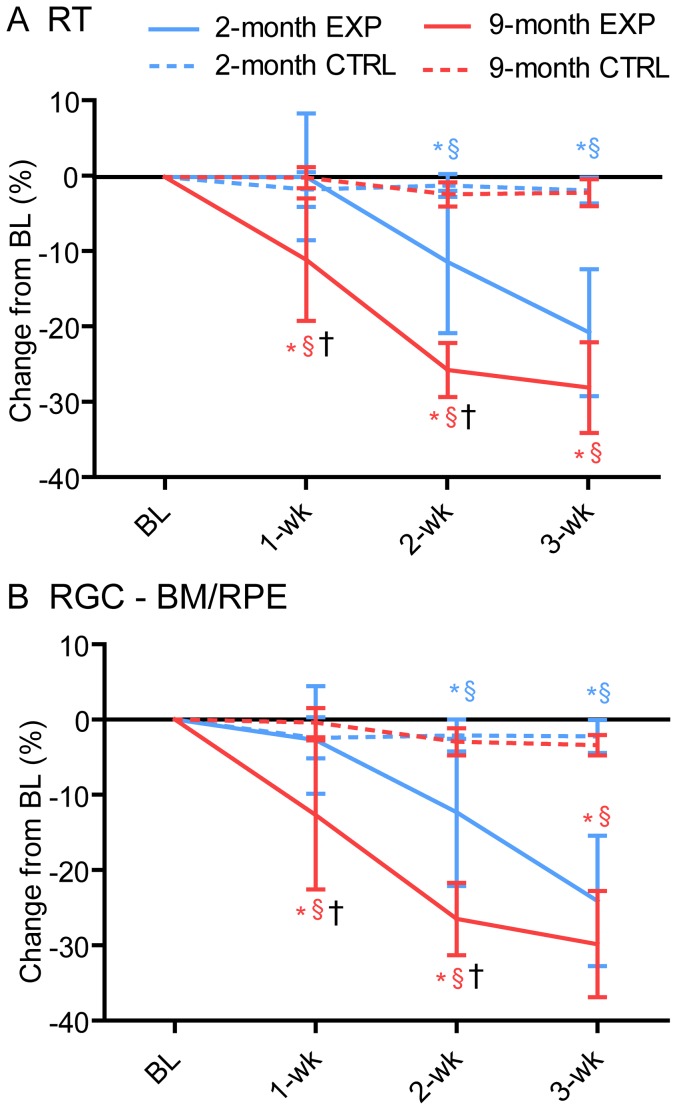
Percent change in RT relative to baseline. (A) Change in total peripapillary RT for experimental and fellow control eyes in both age groups. The 2-month group experimental eyes exhibit no change in thickness at 1-week, followed by thinning increasing over 2- to 3-weeks, while the 9-month group experimental eyes exhibit increasing thinning from 1- to 3-weeks, The 2 age groups show a similar rate of thinning once it begins, however the 9-month group has greater total thinning because change begins at an earlier time point. (B) Change in total retinal thickness excluding the RNFL (i.e.RGC to BM/RPE layers). Results over the 3-weeks follow up show thinning in experimental eyes similar to the results for total retinal thickness. Error bars  =  SEM. * indicates p<0.05 when compared to baseline, § indicates p<0.05 when compared to fellow control eyes, † indicates p<0.05 when comparing 2-month and 9-month experimental eyes (Bonferroni). Abbreviations: EXP  =  experimental eyes, CTRL  =  fellow control eyes, BL  =  baseline.

Total RT was measured from the ILM to the Bruch's membrane/retinal pigment epithelium (BM/RPE) complex at shown in Fig. 2. In the 2-month group experimental eyes, RT was 220.5±4.5 µm at baseline, stable at 1-week (221.3±19.7 µm, p>0.05), then showed progressive thinning relative to baseline at 2-weeks (196.3±19.2 µm; −11.2±9.4%, p<0.001) and 3-weeks (174.8±21.1 µm; −20.6±8.4%, p<0.001). The percent change in RT relative to baseline is shown in Fig. 4A. For RGC to Bruch's membrane/RPE thickness (RT minus RNFL, Fig. 4B) there was also a gradual progression of thinning relative to baseline (1-week: −2.7±7.1%, p>0.05; 2-weeks: −12.3±9.8%, p<0.001; 3-weeks −24.1±8.7%, p<0.001). This indicates that the RNFL thickening at 1-week in 2-month animals is an isolated effect and the other retinal layers are not affected. However, at 2- and 3-weeks there is actually greater relative thinning in the other retinal layers (12–24%) than in the RNFL (7–8%). In the 9-month group experimental eyes (Fig. 4A), RT began 3.8% thinner at baseline (212.2±10.5 µm) than the 2-month group, and decreased progressively after chronic IOP elevation (1-week: 187.4±21.7 µm, −10.9±8.1%, p<0.01; 2-weeks: 157.7±11.5 µm, −25.5±3.6% p<0.001, 3-weeks: 155.6±17.4 µm, −27.8±6.0%, p<0.001). There was no difference in RT between age groups at baseline or at 3-weeks (p>0.05), however the older group showed significantly thinner RT at 1- and 2-weeks (p<0.05) than the younger group, which is due to the different RNFLT changes. There was also progressive thinning relative to baseline of the retina distal to the RNFL, from the RGC layer to Bruch's membrane/RPE (Fig. 4B, 1-week: −12.7±9.9%, p<0.001; 2-weeks: −26.5±4.8%, p<0.001; 3-weeks: −29.8±7.1%, p<0.001). Hence, thinning of both the RNFL (21–22%) and distal layers (RGC to BM/RPE, 26–30%) are contributing to the total retinal thinning. All fellow-control eyes remained stable for RT across the 3-weeks (all p>0.05).

### Anterograde Axonal Transport Assay

**Figure 5:**
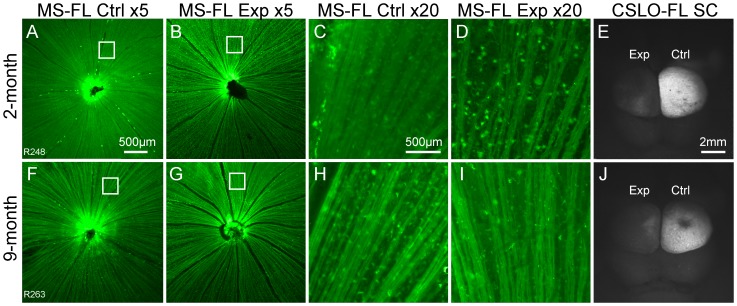
Anterograde axonal transport assay 3-weeks after chronic IOP elevation. The experimental eye results from one representative animal in the 2-month (A-E) and 9-month (F-J) age groups. Post-mortem fluorescence microscopy (MS-FL) images of the ocular fundi from the control (Ctrl; A,F) and experimental (Exp; B,G) eyes 24 h after bilateral intravitreal injections of CTB demonstrate successful CTB uptake and transport by RGCs along their axons within the RNFL to the optic disc. Higher power MS-FL images corresponding to the boxed regions show healthy, straight RGC axons in the control eyes (C,H) and wavy axons in the experimental eyes (D,I). Post-mortem CSLO-FL images of the superior colliculi (E,J) show much greater intensity and coverage of CTB fluorescence in the control side of the colliculus, indicating anterograde axonal transport to the colliculus is intact along the control pathway, but severely disrupted in the experimental pathway in both age groups. Quantitative results for the entire group are shown in Table 1. Scale bar in (A) applies to (A,B,F,G); in (C) applies to (C,D,H,I); in (E) applies to (E,J).

Fig. 5 shows a representative example of anterograde axonal transport assay results at 3-weeks follow-up. RNFL fluorescence 24-hours after bilateral intravitreal CTB injections demonstrates that injections were successful (Fig. 5 A,B,F,G). Higher resolution microscopy (Fig. 5 C,D,H,I) shows fluorescent RGC somas and axons, indicating that CTB uptake and transport within the retina remained intact for many remaining axons. The nerve fibers are straight and arranged in orderly bundles in the control eyes, but show some waviness and bundle-drop out in the experimental eyes. Post-mortem CSLO imaging of the superior colliculi (dorsal midbrain, Fig. 5 E & J) shows higher intensity and greater coverage of CTB fluorescence in the experimental compared to the fellow control pathways. There is a striking difference in the degree of apparent transport failure to the SC considering that RGCs and axons of the experimental eye exhibited relatively preserved uptake and initial transport of CTB within the retina in both young and old animals. Quantitative results for anterograde axonal transport are shown Table 1 and indicate a severe disruption in the experimental pathway after 3-weeks of chronic IOP elevation in both 2-month and 9-month age groups, as shown by the individual examples in Fig. 5. There was no difference in the transport deficit between age groups (p = 0.55). Similar levels of axonal transport blockade were also found in the 2-month old (−62%, n = 1) and the 9-month old (−42%, n = 1) rats with assays performed at 2- and 1-week respectively.

**Table 1:** Results of anterograde axonal transport assay at 3-weeks.

Age group	Exp SC Intensity (±SD a.u.)	Ctrl SC Intensity (±SD a.u.)	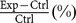	P value (Bonferroni)	N
**2-month**	59.0±20.7	130.2±42.8	−50.4±21.6	<0.001	10
**9-month**	51.9±7.7	124.4±39.8	−52.6±20.7	<0.001	10

Abbreviations: superior colliculi (SC), experimental eye (Exp), fellow control eye (Ctrl).

### Proximal Optic Nerve Grading

**Figure 6:**
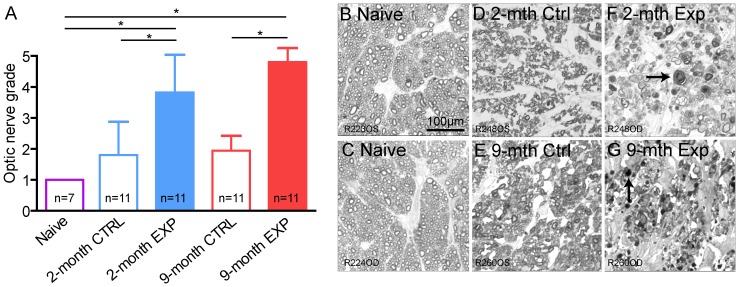
Optic nerve grading. Optic nerve axon damage was assessed by masked, semi-quantitative grading [54], [55] of sections obtained 1 mm from the globe. (A) Results of optic nerve axon grading. (B-G) Representative examples of optic nerve cross-sections at 3-weeks stained with p-phenylenediamine. (B, C). Naïve normal (grade 1). (D) 2-month fellow control (grade 1.5). (E) 9-month fellow control (grade 1.5). (F) 2-month experimental (grade 4). (G) 9-month experimental (grade 5). Arrows point to unraveled myelin seen only in experimental nerves. Both experimental age-groups show severe axon damage relative to fellow control and naïve nerves. Fellow control nerves also show some focal degeneration/swelling relative to the naïve nerves but no unraveling of myelin. Error bars  =  SEM. * indicates p<0.05 (Dunn's post-hoc test). Abbreviations: EXP  =  experimental eyes, CTRL  =  fellow control eyes.

Fig. 6 shows the optic nerve grading results for each group as well as representative examples of optic nerve cross-section images. The naïve normal nerves had a grade of 1 as expected (Fig. 6 A-C). Experimental nerves in both age groups showed significantly greater axon damage with unraveled myelin, thickened septae and gliosis (and thus a higher grade of damage) than both fellow control and naïve normal nerves (p<0.0001; Fig. 6 A, D-G), and a trend for greater severity of damage in the 9-month group. Interestingly, the fellow control nerves also showed a low level of axon degeneration (less than grade 2), which may indicate low grade CTB toxicity (Fig. 6 A,D,E).

### Comparing RNFLT, Axonal Transport and Optic Nerve Axon Damage

**Figure 7:**
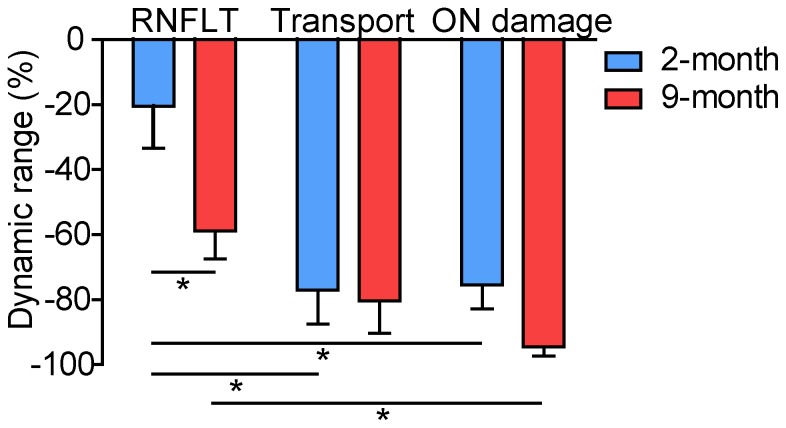
RNFLT, axonal transport and optic nerve damage relative to dynamic range after 3-weeks of unilateral IOP elevation. Loss of RNFLT was substantially less than axonal transport disruption and optic nerve damage and was greater in the younger rats despite a similar IOP insult in both age groups. Error bars  =  SEM. * indicates p<0.05 (Bonferroni post-hoc tests). Abbreviations: ON  =  optic nerve.

The amount of RNFL thinning, axonal transport deficit and optic nerve axon damage were determined relative to their dynamic range in order to compare them against each other on more common grounds (Fig. 7). The maximum relative loss (or “floor”) of the RNFLT measurement was determined from a previous study in rats with unilateral optic nerve transaction [49] and found to be 36% loss from baseline (−36%) when there is approximately 100% RGC loss (soma drop-out). The floor of the axonal transport dynamic range (−65%) was determined from unilateral intravitreal CTB injections in normal rats at a 24 hour assay time-point in a previous study [50] as the normalized percentage difference in fluorescence intensity between superior colliculi. The floor of the optic nerve axon damage was determined from grade 5 representing nearly 100% axon damage (axon loss). Comparing the deficits relative to dynamic range revealed two important findings (Fig. 7). First, the degree of axonal transport disruption and optic nerve damage was worse than the loss of RNFLT measured *in vivo* after 3-weeks of chronic IOP elevation in both age groups (although the difference between transport disruption and RNFLT for the old age group was not statistically significant). This finding is consistent with the individual examples presented in Figs. 5 and 6. Second, the discrepancy between the degree of RNFLT loss relative to axon transport deficit or optic nerve damage was greater in the younger, 2-month group of rats despite a similar magnitude and course of IOP insult between age groups.

## Discussion

This study compared outcomes (*in vivo* RNFLT, axonal transport and optic nerve damage) between young (2-month-old) and older rats (9.5-month-old) during and after a 3-week period of chronic IOP elevation. To our knowledge, this is the first report comparing *in vivo* RNFLT to axonal transport and structural changes in optic nerve axons after extended IOP elevation for longer than 8-hours and the results showed clear differences between outcome parameters and age groups. First, the degree of axonal transport disruption and optic nerve damage were both substantially worse than predicted from the quantitative measure of RNFLT or from the appearance of the RNFL in post mortem flat mounts. The degree of axonal transport deficit at 3-weeks was consistent with the level of optic nerve axon damage, but there was greater preservation of the RNFL in the younger rats. This suggests when early RNFL loss is seen clinically by OCT, there may already be severe axonal transport deficits and even degeneration of optic nerve axons. It also aligns with evidence that the first point of damage to axons is within the ONH [2], [59], [60], with faster anterograde than retrograde damage, since the apparent axonal transport deficit and morphological signs of axon damage were worse within the orbital optic nerve than in the retina. A longitudinal study in monkey has also shown that substantial optic nerve axon loss precedes RNFLT loss [61]. Similar findings have also been reported for other rodent models of glaucoma, including DBA/2J mice with chronic hereditary bilateral glaucoma [10], [12]–[14], [17], [62], IOP elevation induced by laser photocoagulation of the trabecular meshwork [15], [16], [18], [19], and by intracameral microbead injections [17], in which signs of axon degeneration are observed at the earliest stages within the ONH (or even more distally) [17], [62] and progress from distal to proximal segments, with RGC somas and proximal axon segments persisting long after axon segments within and distal to the ONH have degenerated.

The second major finding was that the RNFLT changes were variable with age (Figs. 2–3 and 7) despite similar elevations in IOP (Fig. 1) and evidence of similar distal optic nerve injury (i.e. axonal transport deficit (Figs. 5 and 7)). This indicates the RGC soma and the proximal portion of their axons within the RNFL remains intact longer after IOP injury in younger rats. Previous studies have shown that spontaneous optic nerve axonal degeneration increases exponentially with age [63], [64], but also that IOP elevation and sham cannulation (repeated trauma) cause greater RGC functional loss in older eyes [65], [66]. Specifically, four repeated injections separated by a week have been found to cause changes in retinal function in 18-month old rats but not in 3-month old rats [66]. Furthermore, sham cannulation in 2-month old rats does not have a lasting effect on RNFLT [23]. These studies suggest that repeated injections themselves have a minimal effect on the results in this study. Crish et al [17]. have reported a higher prevalence of degenerating axon profiles in the distal portion of the orbital optic nerve as compared with the proximal portion (nearer the globe) in aging DBA/2 J mice and a tendency for greater persistence of intact proximal axon profiles in the younger mice. Crish et al [17]. also demonstrated that older rats suffered a substantially worse deficit of anterograde axonal transport than younger rats exposed to a similar level of chronic IOP elevation by intracameral microbead injection, however, that report did not include assessment of the relative degree of axon damage/loss by histology or *in vivo* measurements of RNFLT in that group. Collectively, these results indicate the RGCs and axons of older eyes are more susceptible (and younger eyes more resilient) to injury. However, it is also possible the biomechanics of older eyes produce greater injury to axons, glia or blood flow within the ONH as compared with the same amount of IOP insult in younger eyes [3], [67]–[69].

The finding of initial RNFL thickening in the young rats and not in the old rats points to a difference between age groups in the response of RNFL tissues to chronic IOP elevation. Previously, we observed a similar response of RNFL tissue following 8-hours of acute IOP elevation to 50 mmHg in young rats: the RNFL was 11% thicker 1-day after IOP elevation, increasing to a peak at 3–7 days that was 15–19% thicker and resolving by 3-weeks back to baseline, with no loss of RGCs or evidence of RNFL thinning at 6-weeks [23]. It has also been shown in (treated) acute angle closure human patients (IOP >40 mmHg, age 58.48±12.03 years) that transient RNFL thickening occurs and peaks at 3-days (18% relative to fellow control eyes), which is then followed by progressive RNFL thinning (up to 23%) over 1–6 months [70]. Interestingly, a small degree of RNFL thickening (2.4%, p<0.0001) has also been observed in the early stages of experimental glaucoma in non-human primates with unilateral chronic IOP elevation, which is ultimately followed by progressive RNFL thinning [71]. Hence, there is a precedent for the initial thickening observed in the young rats, but intriguing why it is not evident in the older rats. The IOP was actually slightly higher in the older group at 1-day (but still less than in the acute studies), and was equivalent between the two age groups from 3- to 7-days (Fig. 1), hence it is not related to IOP differences. It is possible the sequence of RNFLT change in the young rats (thickening followed by thinning) also occurs in the older rats, but manifests a faster course whereby thickening may have recovered before the 1-week follow-up time point. It is also possible that transient RNFL thickening doesn't occur at all in older rats and they simply progress directly to a phase of response characterized by RNFL thinning. Given that initial RNFL thickening was seen only in the younger rats, and that ultimately there was less RNFL thinning at 3-weeks in the younger rats, the transient RNFL thickening response may even be important in protecting RNFL axons and their somas from degeneration and death, or at least offsetting some amount of RNFL thinning that would otherwise manifest. It remains unclear what the underlying pathology of the RNFL thickening represents, and to what extent an inflammatory or glial response contributes [72], [73]. However, we found previously that RNFL thickening subsequent to 8 hours of IOP elevation does not occur in conjunction with RNFL microglial activation [23].

There are three limitations in this study that warrant discussion. First, as our peak IOP results show (average peak of 61 mmHg, range 47–72 mmHg), many rats experienced IOPs in the range where ischemia might occur, despite the average IOP across the entire 3-week period being 37–38 mmHg. Ischemia occurs at perfusion pressures lower than 25–30 mmHg or IOPs above 70 mmHg [29], [74], [75]. Mean blood pressure in awake normal rats is in the range of 90–105 mmHg [76], hence perfusion pressure may have been as low as 18 mmHg in a few rats for a short time. Furthermore, we noted small hyphemas in 7 of 11 young animals and 2 of 11 older animals at 3-weeks, suggesting neovascularization of the iris due to retinal ischemia. That younger rats may have developed secondary neovascular glaucoma more readily than older rats might explain aspects of the different responses between age groups. However, it is important to note that hyphema was observed first at 19- to 21-days, when the difference in RNFL changes between age groups was already manifest for >2 weeks.

Secondly, there are two factors that might have also contributed to an overestimate of retinal thinning after IOP elevation. The mechanical effect of IOP elevation itself causes changes in the conformation of the globe, especially in and immediately adjacent to the ONH, which results in presumed ‘stretch’ (Poisson compression) of the peripapillary retina and decreased thickness [48], [77], [78]. Also, chronic IOP elevation caused axial elongation myopia, which both stretches the retina and/or confounds thickness measurements due to changes in lateral magnification. Previous studies have shown that dense volume SD-OCT scans combined with individual biometry measures can assist with interpolating results to account for lateral magnification differences [79], [80]. Specifically in rat, changes in axial length of 2 mm (i.e. 33% increase or worse-case scenario) can cause a 2.5 µm change in RNFLT [80], which is substantially less than the changes reported in the current study. Additionally, since circle scans are taken at a fixed angular distance, it is possible that a slightly different peripapillary linear distance from the optic nerve head was assayed in the different age groups due to small differences in biometry; however, the critical result in this comparison is their relative difference in longitudinal course rather than differences at baseline between groups (Fig. 3). In this regard, the fellow eye serves as an important control since both IOP and RNFLT in the fellow eye did not change relative to baseline over follow up in either age group.

Finally, it should be noted that the image segmentation used in this study to determine RNFLT included the major blood vessel profiles, which results in an offset above zero for the lower limit of the dynamic range. Measurements of RNFLT made between the vessels, such as done in a study by Nagata et al [81], would exclude this offset and any effects due to changes in vessel caliber. However, we did not qualitatively observe any obvious vessel changes in contrast to the dramatic changes within the adjacent tissue (Fig. 2).

Axonal transport deficits are found within hours after IOP elevation and are potentially reversible before permanent injury occurs [8], [22], [23]. A previous rodent study showed that IOP elevation to 50 and 75 mmHg for 6 hours reduces axonal transport by 74% and 83% respectively [9]. The previous study by Crish et al. showed an average transport deficit of 60% in 7–9 month-old rats after 13 days of IOP elevation to an average of 28 mmHg [17]. The present study showed a 77–80% axonal transport deficit relative to dynamic range in RGCs at 3-weeks, which correlates to the 75–95% optic nerve axon damage at 3-weeks (Fig. 7). The transport deficit in this study was slightly worse than the deficit in the Crish et al. study, most likely because the average IOP was slightly higher and the duration was 1 week longer. It seems likely the axonal transport deficit increases quickly (over hours) with IOP elevation, and stays at a similar level after permanent injury to the optic nerve, despite IOPs regressing back to a mean of 34 mmHg (young) and 22 mmHg (old) by 3-weeks. Ideally, development of axonal transport assays that enabled repeated longitudinal assessment within the same animals would help determine with even finer temporal resolution the relative course of changes in axonal transport and RNFLT.

The optic nerve axon grading showed severe damage in experimental nerves proximal to the globe (grade 3.8 young, 4.7 old) and also a much milder injury in fellow control nerves (grade 1.8 young, 1.9 old), consisting of axonal swelling and disorganization, but no unraveled myelin (Fig. 6). Optic nerves from naïve normal rats showed no sign of injury (grade 1.0). The fellow control eyes did not have intracameral injection or IOP elevation, but did have intravitreal CTB injection. A previous study using chronic elevation of IOP (hypertonic saline model) in rats shows no axonal damage on optic nerve grading in all untouched fellow eyes (grade 1.0) [54]. Hence, it seems likely the CTB injection has caused the low level axonal swelling and disorganization seen in the fellow control eyes, which indicates a mild CTB toxicity, especially as the RNFL axons are in good condition. A previous study using *in vivo* manganese-enhanced magnetic resonance imaging showed a similar effect of intravitreal manganese on mouse optic nerve axons [82]. CTB is reportedly the non-toxic subunit of cholera toxin and is even used as a vaccine in humans [83]. However, our laboratory has also shown a CTB reaction, as indicated by extensive RNFL thickening and microglial activation, to be present in the fellow eye of rodents with unilateral optic nerve transaction [49]. RNFL thickening was not present in control animals (unilateral sham surgery with CTB or unilateral transection surgery without CTB); hence, the CTB reaction might be limited to only fellow eyes from animals with contralateral optic nerve injury. Importantly, the possibility that CTB is mildly toxic has minimal effect on the present study since the optic nerve and axonal transport results show a major difference between experimental and control pathways and because the RNFLT results were taken before the CTB was injected.

Previous studies have shown that IOP elevation to 30–50 mmHg causes rapid changes in blood flow (within seconds) [84], deformation of the ONH and peripapillary tissues (within minutes) [48], reduction in RGC function by electroretinogram (within minutes to hours) [85], [86], axonal transport deficit (within hours) [8], [9], [22] and RNFL thickening (by day-1) [23]. Recovery after IOP normalization is thought to depend on the extent and length of IOP elevation (IOP-time integral) [29] and also the number of IOP spikes [24]–[28]. There is reversal of all aforementioned changes after acute IOP elevation to 50 mmHg for up to 8 hours; blood flow (within minutes) [84], RGC function (by day-1) [87], axonal transport (by day-7) [23], and RNFL thickening (by 3-weeks) [23]. Although 8 hours of 50 mmHg IOP did not have a permanent effect on axonal transport, RNFLT or RGC death in young rats [23], chronic IOP elevation (mean IOP 37 mmHg) caused severe axonal transport deficits in young and old rats and mild (young) to moderate (old) RNFL thinning after 3-weeks, with RNFL thinning lagging behind optic nerve axon damage. A study using intermittent elevations of IOP to 30–35 mmHg for 1-hour for either 10 or 30 sessions over 6 weeks also showed less retinal thinning than loss of RGC somas or loss of optic nerve axon density [28]. However another study with intermittent elevations of IOP to 35 mmHg for 1-hour, 6-days a week showed greater RNFLT thinning than loss of RGC somas or loss of optic nerve axons, although this study measured RNFLT by histology rather than SD-OCT [27].

## Conclusions

This study found axonal transport disruption and optic nerve damage after 3-weeks of chronic IOP elevation is substantially worse than thinning of the RNFL or changes in its post mortem appearance, indicating relative preservation of the intraretinal portion of RGC axons. This suggests the injury likely occurs within the ONH, with distal axonal damage and transport deficits far exceeding proximal damage and degeneration. This discrepancy was greater in 2-month old rats as compared with 9.5-month old rats, despite a similar course of chronic IOP elevation, indicating an age-related difference in the susceptibility (or resilience) of axons and/or in the ONH biomechanics to this injury. This may be relevant to human glaucoma since increased age is a well-known risk factor for both the onset and progression of glaucomatous damage to the human visual system. The underlying mechanisms are currently under study in both species.
